# Latent tuberculosis infection among patients with and without type-2 diabetes mellitus: results from a hospital case-control study in Atlanta

**DOI:** 10.1186/s13104-021-05662-0

**Published:** 2021-06-30

**Authors:** Argita D. Salindri, J. Sonya Haw, Genet A. Amere, Joyce T. Alese, Guillermo E. Umpierrez, Matthew J. Magee

**Affiliations:** 1 Division of Epidemiology and Biostatistics, Department of Population Health Sciences, School of Public Health, Georgia State University, Atlanta, GA 30303 USA; 2 Division of Endocrinology, Metabolism and Lipids, Emory University School of Medicine, Atlanta, GA USA; 3 Hubert Department of Global Health, Rollins School of Public Health, Emory University, Atlanta, GA USA

**Keywords:** Tuberculosis, Type-2 diabetes, Latent TB infection

## Abstract

**Objective:**

The purpose of this study is to compare the prevalence of latent TB infection (LTBI) among patients with type-2 diabetes mellitus (T2DM) to healthy controls without T2DM. To achieve this objective, we conducted a case-control study in a large hospital in Atlanta from 2016 to 2019.

**Results:**

We enrolled 98 cases; 119 potential controls were screened, 84 of which had HbA1c  ≥  5.7% and one did not have QFT result, leaving 34 (28.6%) individuals enrolled as controls. LTBI prevalence was 9.2% among cases and 14.7% among controls (crude odds ratio 0.59, 95% CI 0.19–2.04). After adjusting for age and sex, the adjusted odds of LTBI among patients with T2DM was 0.45 (95% CI 0.13, 1.71) times the controls. We did not observe a statistically significant association between LTBI and T2DM. However, we reported a positive correlation between HbA1c level and nil count among individuals with LTBI (R^2^  =  0.55, p  <  0.01). In addition, we reported a high prevalence of LTBI among adults with T2DM and family members without T2DM.

**Supplementary Information:**

The online version contains supplementary material available at 10.1186/s13104-021-05662-0.

## Introduction

Emerging cross-sectional evidence suggests type 2 diabetes mellitus (T2DM) is associated with higher prevalence of latent tuberculosis infection (LTBI) [1, 2]. Results from US national survey data reported 12% LTBI prevalence among adults with diabetes compared to 5% LTBI prevalence among adults without diabetes [3, 4]. However, less is known regarding US regional differences in the relationship between LTBI and T2DM or the extent that the association is different in racial/ethnic subgroups [5].

Although T2DM is an established risk factor for tuberculosis (TB) disease, whether T2DM increases the risk of LTBI, or vice versa, remains a critical gap in knowledge [2, 6]. Results from murine models of diabetes and TB suggest that phagocytosis and uptake of *Mycobacterium tuberculosis* (*Mtb*) by monocytes and macrophages are reduced in the context of diabetes [7]. Pro-inflammatory cytokine expression profiles associated with *Mtb* control [i.e., interferon (IFN)-γ] were also delayed in diabetic animal models [8]. These may support the premise that diabetes increases the risk of TB infection or reactivation from latency. Alternatively, LTBI may influence T2DM risk as increasing evidence indicates TB modulates human adipose tissue function and may impact host metabolic homeostasis [9]. For example, an animal model of nondiabetic Guinea pigs reported that an infection of *Mtb* alone could result in glucose intolerance and incresead level of serum free fatty acid, two strong predictors of T2DM development [10].

To definitively establish whether LTBI increases diabetes risk or impact host metabolic outcomes will require large longitudinal studies and extensive follow-up time. Thus to gain preliminary insight into the LTBI-T2DM relationship, we conducted a case-control study to (a) compare the prevalence of LTBI among patients with T2DM to controls without T2DM, and (b) assess whether glycated hemoglobin (HbA1c) level is correlated with QuantiFERON Gold-in-tube test (QFT) quantitative measures (i.e., antigen, mitogen, or nil count).

## Main text

### Methods

We conducted a case-control study in a large hospital in metro Atlanta, Georgia, US, from 2016 to 2019. Eligible cases were human immunodeficiency virus (HIV)-negative adults (≥ 21 years) with newly diagnosed T2DM (diagnosed within the past 3 years) and no history of TB disease. Among cases, HbA1c (%) was obtained from the electronic medical chart by abstracting the plasma HbA1c value closest to the date of study enrollment. Eligible controls included adult family members/friends of cases with HbA1c  <  5.7% (measured at the time of screening by a point-of-care HbA1c [11], Siemens DCA Vantage Analyzer) and no self-reported prior diagnosis of pre-diabetes, T2DM, HIV, or TB disease. HbA1c values were categorized according to the American Diabetes Association classification with HbA1c  <  5.7% considered as “normoglycemic”, HbA1c 5.7–6.4% as “pre-diabetes” and, HbA1c  ≥  6.5% as “diabetes” [12]. Enrolled participants had LTBI status determined by QFT Gold-In-Tube test at the time of study enrollement. QFT samples were prepared and processed following the manufacturer’s (QIAGEN) guidelines. Results of the quantitative QFT measures were interepreted using three ciriteria: (a) mitogen-nil and tuberculin-nil values, (b) percentage of avian difference, and (c) percentage of tuberculin response [13], and classified as “positive”, “negative”, or “indeterminate” for TB infection. Cases and controls were excluded if they were using steroids or tumor necorosis factor (TNF)-α antagonist therapy at the time of screening, resided outside DeKalb or Fulton counties, or did not speak English. Participants clinical and demographic characteristics were obtained using study questionnaires and medical chart review.

#### Data analysis

We compared demographic and clinical characteristics of cases to controls using Chi-square and Fisher’s Exact tests. Logistic regression was used to estimate the association between LTBI and T2DM quantified by odds ratios and 95% confidence intervals (CIs). Covariates included in the final adjusted model were selected purposively based on previously published literature and directed acyclic graph theory [14]. Among those with LTBI, linear regression was used to estimate the correlation between HbA1c and quantitative QFT measures. Linear regression models were used to measure the relationship between HbA1c and (a) nil count, (b) TB antigen-nil, and (c) mitogen-nil values. In addition to beta estimates and corresponding 95% CI, we also reported R^2^ values or the coefficient of determination as a measure of how well the linear regression model described the observed data.

## Results

We screened 199 potential eligible cases and 405 potential eligible controls during the study period. Among individuals screened, a total of 98 cases and 34 controls were enrolled. Of 199 eligible cases, 91 (45.7%) refused to participate or were excluded (Additional file 1: Figure S1). Out of 108 cases enrolled, 10 were later excluded, leaving 98 (90.7%) included in the final analyses. Among 405 potential eligible controls, 35 (12.2%) self-reported prior diagnosis of pre-diabetes/T2DM, 251 were either refused to participate or excluded. We screened 119 (29.3%) potential eligible controls with HbA1c test, and 29.4% (35/119) had HbA1c  <  5.7%, 34 were included in the final analyses. Among screened controls who were excluded (n  =  85), the median HbA1c was 6.0 [interquartile range (IQR) 5.8–6.2]. The majority of our study participants were African American (92.9% among cases, 79.4% among controls; Table 1). Cases were older (median age  =  54, IQR 49–60) than controls (median age  =  51, IQR 35–57) (p  =  0.02). Daily smokers were more common among the controls (35.3%) vs. cases (24.7%) (p  =  0.02). The proportions of individuals with previous diagnosis of high cholesterol (70.6% vs. 5.8%) and high blood pressure (70.8% vs. 35.3%), or obesity (66.3% vs. 35.3%) were greater among cases compared to controls (p  <  0.05).

**Table 1:** Demographic and clinical characteristics of BATT study participants, Atlanta, Georgia 2016–2019 (N  =  132)

Characteristics	Controls N = 34	Cases N = 98	Total N = 132	X^2^ p value
LTBI status				
Negative	29 (85.3)	89 (90.8)	118 (89.4)	0.35*
Positive	5 (14.7)	9 (9.2)	14 (10.6)	
Age group				
21–40	13 (38.2)	7 (7.1)	20 (15.2)	**< 0.01**
41–60	15 (44.1)	70 (71.4)	85 (64.4)	
> 60	6 (17.7)	21 (21.4)	27 (20.5)	
Gender				
Male	17 (50.0)	42 (42.9)	59 (44.7)	0.47
Female	17 (50.0)	56 (57.1)	73 (55.3)	
Race/ethnicity				
Non-hispanic white	3 (8.8)	3 (3.1)	6 (4.6)	0.15*
Non-hispanic black	27 (79.4)	91 (92.9)	118 (89.4)	
Hispanic	1 (2.9)	1 (1.0)	2 (1.5)	
Asian or pacific islander	1 (2.9)	1 (1.0)	2 (1.5)	
Other	2 (5.9)	2 (2.0)	4 (3.0)	
Highest education				
Less than high school	8 (23.5)	18 (18.4)	26 (19.7)	0.15*
High school graduate	15 (44.1)	60 (61.2)	75 (56.8)	
College/university	8 (23.5)	18 (18.4)	26 (19.7)	
Graduate school	3 (8.8)	2 (2.0)	5 (3.8)	
Ever lived with TB-sick person				
No	33 (97.1)	89 (93.7)	122 (94.6)	0.67*
Yes	1 (2.9)	6 (6.3)	7 (5.4)	
Not sure	0	3	3	
Ever told to have positive TST				
No	32 (94.1)	85 (88.5)	117 (90.0)	0.51*
Yes	2 (5.9)	11 (11.5)	13 (10.0)	
Not sure	0	2	2	
Current smoking				
Daily	12 (35.3)	24 (24.7)	36 (27.5)	**0.02**
Less than daily	6 (17.7)	5 (5.2)	11 (8.4)	
Not at all	16 (47.1)	68 (70.1)	84 (64.1)	
Don’t know/refused	0	1	1	
Past smoking				
Daily	24 (70.6)	50 (51.0)	74 (56.1)	0.12
Less than daily	3 (8.8)	10 (10.2)	13 (9.9)	
Not at all	7 (20.6)	38 (38.8)	46 (34.1)	
Alcohol consumption				
Never	18 (52.9)	65 (66.3)	83 (62.9)	0.27
Moderate	11 (32.4)	26 (26.5)	37 (28.0)	
Frequent	5 (14.7)	7 (7.1)	12 (9.1)	
Ever diagnosed with high cholesterol level				
No	30 (88.2)	27 (29.4)	57 (45.2)	**< 0.01**
Yes	4 (5.8)	65 (70.6)	69 (54.8)	
Not sure	0	6	6	
Ever diagnosed with high blood pressure				
No	22 (64.7)	28 (29.2)	50 (38.5)	**< 0.01**
Yes	12 (35.3)	68 (70.8)	80 (61.5)	
Not sure	0	2	2	
Ever diagnosed with heart disease				
No	33 (97.1)	80 (84.2)	113 (87.6)	0.07*
Yes	1 (2.9)	15 (15.8)	16 (12.4)	
Not sure	0	3	3	
Ever diagnosed with liver disease				
No	32 (94.1)	90 (96.8)	122 (96.1)	0.61*
Yes	2 (5.9)	3 (3.2)	5 (3.9)	
Not sure	0	5	5	
Ever diagnosed with kidney disease				
No	34 (100.0)	88 (92.6)	122 (94.6)	0.19*
Yes	0 (0.0)	7 (7.3)	7 (5.4)	
Not sure	0	3	3	
Family members with T2DM				
No	7 (20.6)	29 (29.6)	36 (27.3)	0.31
Yes	27 (79.4)	69 (70.4)	96 (72.7)	
BMI				
Normal (18.5–25)	12 (35.3)	11 (11.2)	23 (17.4)	< 0.01*
Underweight (< 18.5)	0 (0.0)	3 (3.1)	3 (2.3)	
Overweight (25–30)	10 (29.4)	19 (19.4)	29 (22.0)	
Obese (≥ 30)	12 (35.3)	65 (66.3)	77 (58.3)	
Glycated hemoglobin level				
Median (IQR)	5.4 (5.2–5.5)	7.3 (6.4–9.5)	6.7 (5.6–8.7)	**< 0.01**

Bold indicates that the finding is significant at α  =  0.05

*BMI *body mass index; *T2DM *type-2 diabetes mellitus; *IQR *interquartile range; *LTBI *latent tuberculosis infection; *TB *tuberculosis; *TST *tuberculin skin test

^*^p value obtained from Fisher’s exact tests

LTBI prevalence was higher among controls without T2DM (14.7%, 5/34), compared to patients with newly diagnosed T2DM (9.2%, 9/98; Table 2). After adjusting for age and gender, the odds of LTBI among cases was 0.45 times the odds among controls (95% CI 0.13–1.71). The median HbA1c was 7.0% among cases with LTBI vs. 7.3% among cases without LTBI (p  =  0.75). The median HbA1c was 5.3% among controls with LTBI vs. 5.4% among controls without LTBI (p  =  0.37). Although non-significant, cases receiving metformin were less likely to have LTBI when compared to cases who were not receiving metformin [odds ratio (OR) 0.44, 95%CI 0.11–1.92].

**Table 2:** Crude and adjusted odds ratio of latent TB infection among BATT study participants, Atlanta, Georgia 2016–2019 (N  =  132)

Characteristics	LTBI status		Total N = 132	cOR (95% CI)	aOR (95% CI)
	Negative N (%) = 118 (89.4)	Positive N (%) = 14 (10.6)			
Group					
Controls	29 (85.3)	5 (14.7)	34 (25.8)	Reference	Reference
Cases (T2DM)	89 (90.8)	9 (9.2)	98 (74.2)	0.59 (0.19–2.04)	0.45 (0.13–1.71)
Age group					
21–40	19 (95.0)	1 (5.0)	20 (15.2)	0.59 (0.03–3.59)	0.40 (0.02–2.75)
41–60	78 (91.8)	7 (8.2)	85 (64.4)	Reference	Reference
> 60	21 (77.8)	6 (22.2)	27 (20.5)	3.18 (0.94–10.61)	3.09 (0.89–10.44)
Gender					
Male	52 (88.1)	7 (11.9)	59 (44.7)	Reference	Reference
Female	66 (90.4)	5 (9.6)	73 (55.3)	0.79 (0.26–2.44)	1.09 (0.34–3.48)
Race/ethnicity					
Non-hispanic black	105 (89.0)	13 (11.0)	118 (89.4)	1.6 (0.28–30.4)	
Other	13 (92.9)	1 (7.1)	14 (10.6)	Reference	
Highest education					
Less than high school	22 (84.6)	4 (15.4)	26 (19.7)	Reference	
High school graduate	70 (93.3)	5 (6.7)	75 (56.8)	0.39 (0.10–1.71)	
College/university	21 (80.8)	5 (19.2)	26 (19.7)	1.31 (0.31–5.93)	
Graduate school	5 (100.0)	0 (0.0)	5 (3.8)	N/A	
Ever lived with TB-sick person					
No	110 (90.2)	12 (9.8)	122 (94.6)	Reference	
Yes	5 (71.4)	2 (28.6)	7 (5.4)	3.67 (0.49–19.20)	
Not sure	3	0	3		
Currently on metformin, among cases (N = 98)					
No	23 (85.2)	4 (14.8)	27 (27.8)	Reference	
Yes	65 (92.9)	5 (7.1)	70 (72.2)	0.44 (0.11–1.92)	
Missing	1	0	1		
Current smoking					
No	76 (90.5)	8 (9.5)	84 (64.1)	Reference	
Yes	41 (87.2)	6 (12.8)	47 (35.9)	1.39 (0.43–4.27)	
Missing	1	1	2		
Past smoking					
No	44 (97.8)	1 (2.2)	45 (34.1)	Reference	
Yes	74 (85.1)	13 (14.9)	87 (65.9)	7.73 (1.46–142.75)	
Alcohol consumption					
Never	75 (90.4)	8 (9.6)	83 (62.9)	Reference	
Moderate	32 (86.5)	5 (13.5)	37 (28.0)	1.47 (0.42–4.74)	
Frequent	11 (91.7)	1 (8.3)	12 (9.1)	0.85 (0.04–5.34)	
Ever diagnosed with high cholesterol level					
No	50 (87.7)	7 (12.3)	57 (45.2)	Reference	
Yes	62 (89.9)	7 (10.1)	69 (54.8)	0.81 (0.26–2.50)	
Not sure	6	0	6		
Ever diagnosed with high blood pressure					
No	47 (94.0)	3 (6.0)	50 (38.5)	Reference	
Yes	69 (86.3)	11 (13.8)	80 (61.5)	2.50 (0.73–11.48)	
Not sure	2	0	2		
Ever diagnosed with heart disease					
No	101 (89.4)	12 (10.6)	113 (87.6)	Reference	
Yes	14 (87.5)	2 (12.5)	16 (12.4)	1.20 (0.18–5.05)	
Not sure	3	0	3		
Ever diagnosed with liver disease					
No	109 (89.3)	13 (10.7)	122 (96.1)	Reference	
Yes	4 (80.0)	1 (20.0)	5 (3.9)	2.10 (0.10–15.57)	
Not sure	5	0	5		
Ever diagnosed with kidney disease					
No	108 (88.5)	14 (11.5)	122 (94.6)	Reference	
Yes	7 (100.0)	0 (0.0)	7 (5.4)	N/A	
Not sure	3	0	3		
Family members with T2DM					
No	29 (80.6)	7 (19.4)	36 (27.3)	Reference	
Yes	89 (92.7)	7 (7.3)	96 (72.7)	0.33 (0.10–1.03)	
BMI					
Normal (18.5–25)	19 (82.6)	4 (17.4)	23 (17.4)	Reference	
Underweight (< 18.5)	3 (100.0)	0 (0.0)	3 (2.3)	N/A	
Overweight (25–30)	28 (96.6)	1 (3.5)	29 (22.0)	0.17 (0.01–1.26)	
Obese (≥ 30)	68 (88.3)	9 (11.7)	77 (58.3)	0.63 (0.18–2.52)	
Glycated hemoglobin level					
Median (IQR)	6.7 (5.6–8.6)	6.0 (5.3–9.2)	6.7 (5.6–8.7)		

*aOR *adjusted odds ratio; *BMI *body mass index; *CI *confidence interval; *cOR *crude odds ratio; *T2DM *type-2 diabetes mellitus; *IQR *interquartile range

**Fig. 1:**
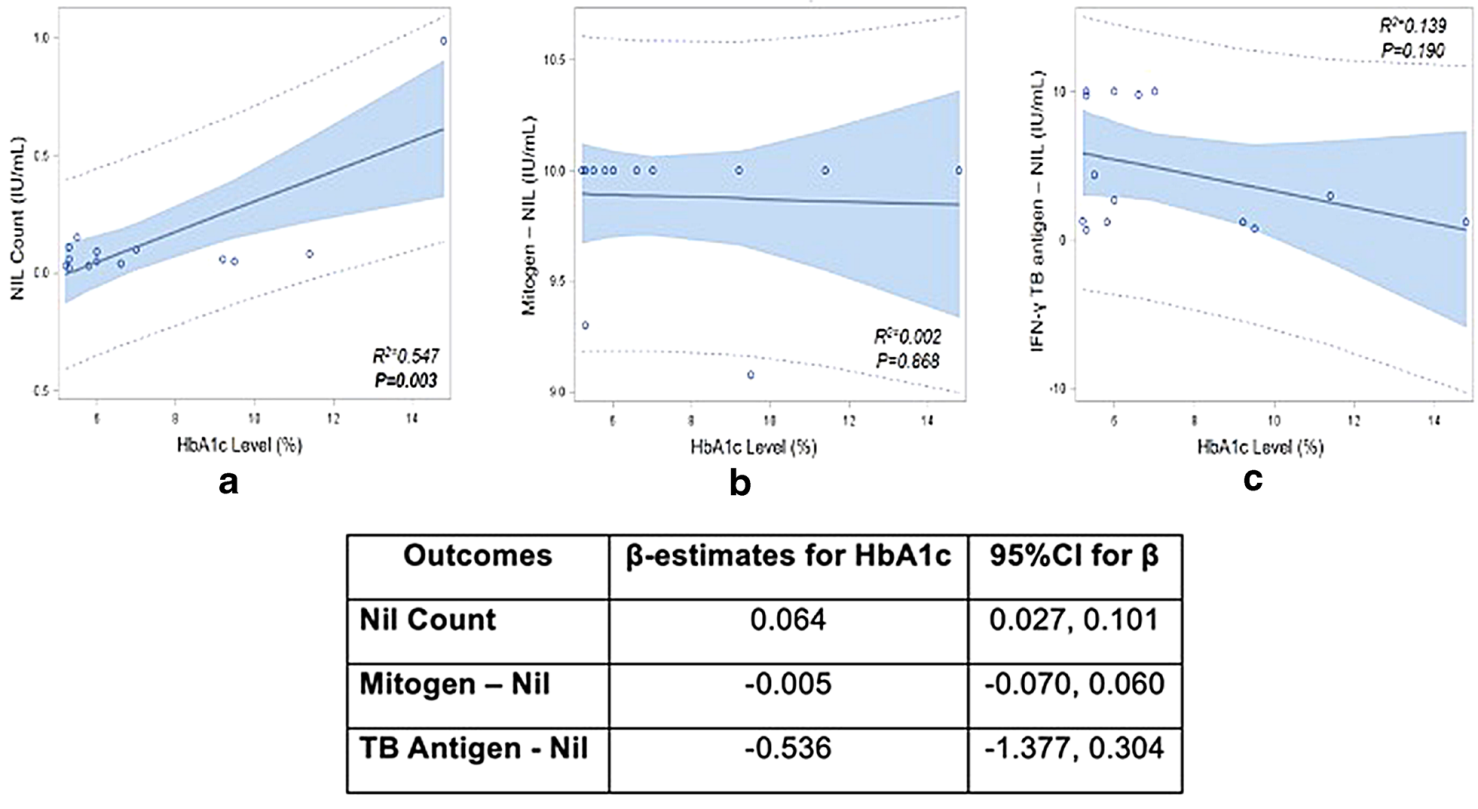
Results from linear regression of nil count, mitogen-nil, and TB antigen-nil by HbA1c level among patients with positive QFT, N  =  14

Among those with LTBI, increasing HbA1c level was correlated with increasing QFT nil values (*R*^*2*^  =  *0.547, p * =  *0.003; *Fig. 1). Among those with LTBI, nil count increased on average by 0.064 (95% CI 0.027–0.101) for every unit increase in the HbA1c level (i.e., every one percentage point increase of HbA1c). We did not observe a significant correlation between HbA1c and TB antigen-nil (*R*^*2*^  =  *0.139, p * =  *0.*190) or mitogen-nil (*R*^*2*^  =  *0.002, p*  =  *0.868*) values. For instance, for every unit increase in HbA1c level, the mitogen-nil value decreased on average by 0.005 (95% CI − 0.070–0.060). Similarly, for every unit increase in HbA1c level, the TB antigen-nil decreased on average by 0.536 (95% CI − 1.377–0.304).

## Discussion

We reported a high prevalence of LTBI among patients with newly diagnosed T2DM and healthy controls without T2DM in the metro Atlanta area. Our LTBI prevalence estimates (9.2% among cases, 14.7% among controls) were higher compared to the US national (5.0%) and US race-specific estimates among African Americans (5.3%) [15]. Inconsistent with our findings, previous studies using nationally representative data from the US reported that diabetes is associated with increased odds of LTBI by one–three-fold [3, 4]. Another study from Atlanta conducted among recently arrived refugees also reported higher LTBI prevalence among participants with diabetes and pre-diabetes compared to euglycemic participants [1]. However, it is important to note that this hospital-based study, conducted primarily among African Americans, is consistent with recent US data that suggests the relationship between LTBI and T2DM differs across racial/ethnic groups [5].

Our findings also suggest that there may be regional differences in the relationship between LTBI and T2DM, which could be affected by background prevalences of both diseases or socioeconomic characteristics (i.e., an established risk factor for LTBI and T2DM) [2]. Of note, we reported more than 70% prevalence of undiagnosed pre-diabetes among screened family members/friends of cases. This finding is consistent with previously published studies reporting high prevalence of prediabetes/diabetes among household/family members of individuals with T2DM [16–18]. Our findings also support previous recommendations suggesting that friends or family members of patients with T2DM would benefit from T2DM screening in an effort to introduce early intervention to prevent T2DM development.

Among individuals with LTBI, we reported a positive correlation between HbA1c and the QFT negative control without antigens or mitogens (nil count). The nil value is used to determine if patient has a pre-existing non-specific immune response which could lead to a false-positive. A cross-sectional study among US adults with LTBI reported a higher IFN-γ antigen response among those with pre-diabetes compared to euglycemic adults, but found that the average nil value was similar among patients with diabetes, prediabetes, and euglycemia [19]. To date, the relationship between the quantitative IFN-γ responses specific to *Mtb* and T2DM is inconclusive. We did not observe a significant correlation between glycemic control with TB antigen or mitogen responses, although we only analyzed this relationship in 14 study participants. Similar to our findings, a cross-sectional study conducted in Indonesia from 2014 to 2015 reported no significant difference of median TB antigen-nil value across different HbA1c levels (HbA1c  <  7.0% vs. 7.0–9.9% vs.  ≥  10.0%; p  =  0.73) [20]. However, a 2014 cross-sectional study conducted in India reported that individuals with LTBI and T2DM had decreased mean of *Mtb* antigen-stimulated (net cytokines) levels including lower IFN-γ (10.5 pg/mL vs. 249.2 pg/mL), TNF-α (6.5 pg/mL vs. 328.1 pg/mL), interleukin (IL)-17A (14.2 pg/mL vs. 24.4 pg/mL), and IL-10 (95.6 pg/mL vs. 220.6 pg/mL) when compared to LTBI individuals without T2DM [21]. Further studies to better characterize the relationship between T2DM, hyperglycemia, and immune responses specific to LTBI are still warranted.

## Conclusion

In conclusion, we reported a high prevalence of LTBI among adults with T2DM and family members without T2DM. Although we did not observe a significant association between LTBI prevalence and T2DM we did observe a positive correlation between HbA1c and nil count among individuals with LTBI. Larger prospective investigations across different regions and race/ethcity subgroups are warranted to determine the role of LTBI in pre-diabetes/T2DM risk. Further studies that measure LTBI prevalence among household members living with patients with T2DM are also needed to determine whether household/family members of individuals with T2DM could be considered as a priority target group for LTBI screening.

## Limitations

Our study was subject to several limitations. First, we had a small sample size enrolled from a single hospital and new diabetes cases were diagnosed within the past three years. Thus, our results may not be widely generalizable to other settings or other new diabetes patients. However, we used 3 years cut-off to define newly diagnosed T2DM due to the long natural progression of T2DM [22, 23] and the potential delay in receiving T2DM diagnosis among our study population, of which the majority came from lower socio-economic levels [24, 25]. Second, our study was designed to assess the association between LTBI and the risk of T2DM. The non-significant findings we reported in this manuscript may in part be due to a bi-directional relationship between LTBI and T2DM where T2DM increases the risk of LTBI. Third, because we were unable to compare at what age cases or controls were exposed to MTB, the higher prevalence of LTBI among controls may be a reflection of when during their lifecourse they were initially infected with LTBI. Last, we enrolled a smaller number of controls compared to cases, which may due to our control selection strategy (i.e., friends or family members vs. community-based recruitment). It is plausible that friends/family members shared lifestyle or other modifiable T2DM risk factors including diet, physical activity, or smoking [18, 26], which could lead to pre-diabetes (HbA1c  ≥  5.7%) or previous T2DM diagnosis and exclusion from this study. Previous studies highlighted that selecting friends/family as controls may pose several epidemiologic challenges including potential case-control overmatch, similarity in responding study’s questionnaires, and potential bias among cases in nominating their controls [27]. However, we believe that using friends or family members as controls is still a viable and cost-efficient option for a small pilot project like the present study.

## Supplementary Information

**Additional file 1: Figure S1.** Study enrollment diagram.

## Data Availability

The datasets generated and/or analysed during the current study are not publicly available due to the confidential nature of patients’ data but are available from the corresponding author on reasonable request.

## Abbreviations

aOR: Adjusted odds ratio
CI: Confidence interval
cOR: Crude odds ratio
HbA1c: Glycated hemoglobin
HIV: Human immunodeficiency virus
IFN-γ: Interferon gamma
IL: Interleukin
IQR: Interquartile range
LTBI: Latent tuberculosis infection
*Mtb*: *Mycobacterium tuberculosis*
OR: Odds ratio
QFT: Quantiferon
T2DM: Type-2 diabetes mellitus
TB: Tuberculosis
TNF-α: Tumor necrosis factor alpha
US: United States of America
