# Supplementary material for: Latent tuberculosis infection among patients with and without type-2 diabetes mellitus: results from a hospital case-control study in Atlanta
Source: BMC Res Notes. 2021 Jun 30;14:252. doi: 10.1186/s13104-021-05662-0 (PMC8247096; doi:10.1186/s13104-021-05662-0)
Supplement: Supplementary file 1 — Additional file 1: Figure S1. Study enrollment diagram. [file 13104_2021_5662_MOESM1_ESM.docx]

Figure S1. Study enrollment diagram


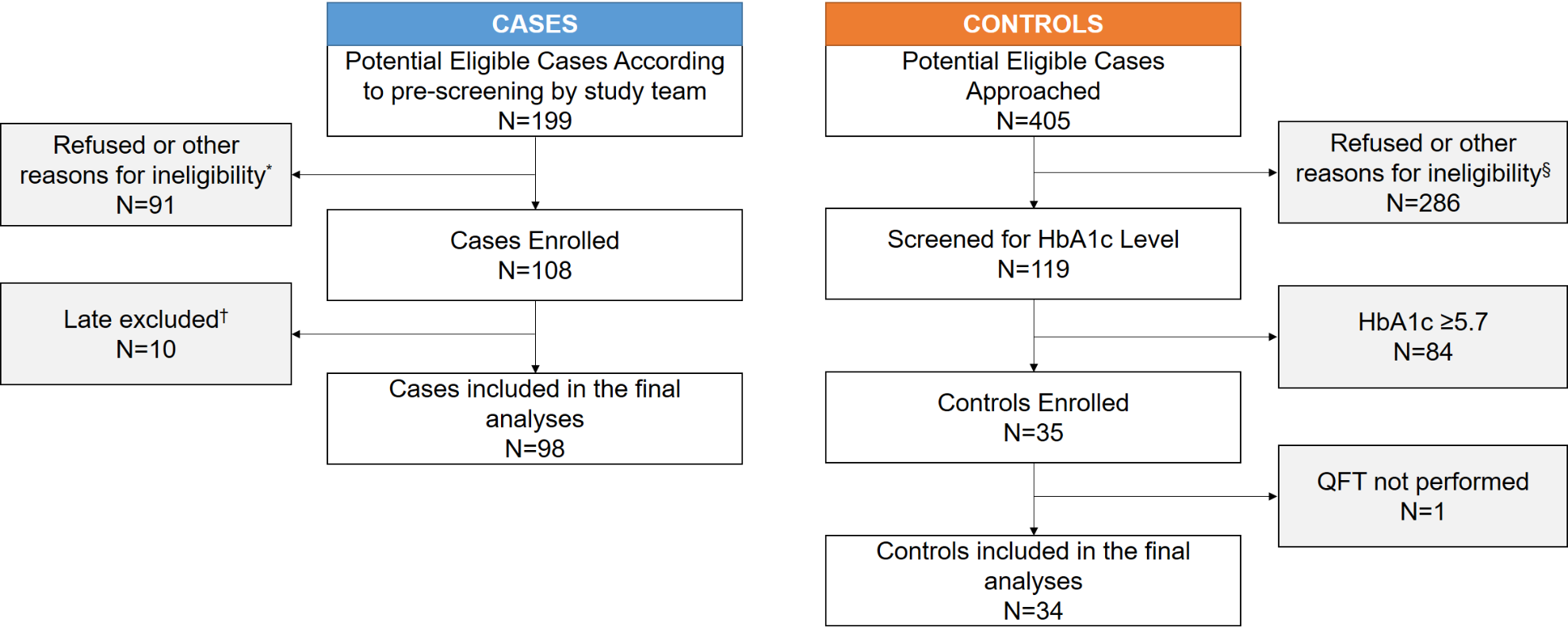


*Including 1 patient who self-reported the use of steroids and 1 patient who was part of another diabetes study

^†^Reasons for late exclusion: 7 had been diagnosed with T2DM for > 3 years (according to medical chart review), 2 were later confirmed as patients with pre-diabetes, and 1 self-reported history of active TB disease during questionnaire interview

^§^Reasons for exclusion among controls (N=286):

- 176 (61.5%) refused
- 36 (12.6%) were not Fulton/DeKalb counties residents
- 35 (12.2%) self-reported prior T2DM/pre-diabetes diagnosis
- 11 (3.8%) were non-English speakers
- 7 (2.4%) were less than 21 years old at the time of encounter
- 4 (1.4%) were HIV positive
- 17 (5.9%) were not enrolled due to other reasons (e.g., were on steroids or other invasive therapy, patients at the sickle cells clinic
